# Medium-Chain Triglyceride Dietary Supplements Reduce Glucose Metabolism of Gait-Related Skeletal Muscle in Older Adults: A Longitudinal ^18^F-FDG PET/CT Analysis

**DOI:** 10.3390/nu17101707

**Published:** 2025-05-18

**Authors:** Tatsushi Mutoh, Hiroki Kataoka, Yasuko Tatewaki, Yasuyuki Taki

**Affiliations:** 1 Department of Aging Research and Geriatric Medicine, Institute of Development, Aging and Cancer, Tohoku University, Aoba-ku, Sendai 980-8575, Japan; 2 Division of Neurocritical Care, Research Institute for Brain and Blood Vessels, Akita Cerebrospinal and Cardiovascular Center, Senshu-Kubota-machi, Akita 010-0874, Japan

**Keywords:** glucose metabolism, gait, ketone, medium-chain triglyceride, elderly

## Abstract

Background/Objectives: Dietary supplementation with medium-chain triglycerides (MCTs) improves walking balance and cognitive function in healthy older adults. This study aimed to determine the biological effects of MCTs on gait-related skeletal muscles in healthy older adults by analyzing muscle density and glucose metabolism. Methods: ^18^F-FDG-PET/CT imaging data from 63 participants (18 g/day of MCTs and matching placebo in the form of a jelly stick [6 g each, ingested 3 times/day]) in a randomized clinical trial were analyzed. The three-dimensional regions of interest were set as muscles associated with walking balance (bilateral triceps, psoas, and vastus medialis). Each muscle’s mean standardized uptake value (SUV_mean_) and Hounsfield units (HU) were calculated for relative quantitative measurements. Results: MCT supplementation for 3 months decreased the SUV_mean_ (*p* < 0.001) and increased the HU of the psoas (*r* = −0.61) and vastus medialis muscles (*r* = −0.59) (*p* < 0.001); no changes were apparent in participants supplemented with long-chain triglycerides. The changes in the SUV_mean_ for each muscle were correlated negatively with those of plasma β-hydroxybutyrate in MCT-supplemented participants (*r* = −0.57 [psoas] and −0.59 [vastus medialis]; *p* < 0.001). Conclusion: A 3-month MCT supplementation suppressed glucose metabolism and increased the muscle density in gait-related skeletal muscles, consistent with previous findings that MCT supplementation stabilizes balance functions during walking in healthy older adults.

## 1. Introduction

Establishing a good health status is an important global issue for an aging population [1]. Among healthy older adults, age-related cognitive and muscle function decline is considered frailty and is a serious societal burden [2,3]. Sarcopenia and dementia are major disorders that shorten life expectancies and increase proportionally with aging [4]. Therefore, preventing these physiological or pathological events before the onset of age-related frailty is especially important.

Ketone bodies are an alternative energy substrate for organs requiring glucose, especially in the older brain, where glucose utilization deteriorates [5]. Medium-chain triglyceride (MCT) oil comprises mixed fatty acids (predominantly the ketogenic compounds caprylic [C8] and capric acids [C10]) extracted from natural products, including milk fat, palm kernel, and coconut oils. In rodents, MCT is an immediate energy source facilitating exercise performance through activating mitochondrial biogenesis and metabolism [6]. MCT supplementation (12-18 g/day) demonstrated positive effects on cognition, both at rest and following exercise in healthy adults [7]. Combining MCTs with aerobic exercise extends its efficacy in improving muscle function and subjective physical and mental health in middle-aged and older adults with poor exercise habits or low body mass index (<24.0 kg/m^2^) at high risk for frailty [8,9]. A recent double-blind, randomized clinical trial (RCT) showed that a 3-month supplementation of MCT oil (18 g/day; 3 meals/day) in healthy older adults improves gait, balance, and executive functions compared with a placebo control comprising long-chain triglyceride (LCT) oil without MCTs [10].

Physiologically, MCTs provide an energy supply to the brain via both direct and indirect (ketone bodies synthesized via β-oxidation in the liver and acylated ghrelin in the stomach) pathways of digested medium-chain fatty acids (MCFAs) [11,12]. Furthermore, MCFAs circulating in the peripheral blood can also act on skeletal muscle to enhance mitochondrial biosynthesis and mitochondrial metabolic activity [11]. However, the biological mechanisms of the MCTs’ essential modulating of the muscle metabolism and function in older adults have not been elucidated.

Skeletal muscle is a primary contributor to energy expenditure because of the substantial amount of energy used to maintain gait and posture to mediate locomotion [13]. ^18^F-2-fluoro-2-deoxy-D-glucose (FDG) positron emission tomography (PET) can quantify the metabolic activity of skeletal muscle, as well as other body organs such as the brain and liver. The PET-derived standardized uptake value (SUV) is proportional to the metabolic activity of the skeletal muscle [14], and thus the mean values (SUV_mean_) in each region of interest (ROI) analyzed by computed tomography (CT) scan enables assessment of energy expenditure of the specific muscle area. In addition, muscle quality assessment using CT scans involves analyzing the density of skeletal muscle, measured in Hounsfield Units (HU), within specific ROIs [15]. This CT-derived skeletal muscle density provides insights into muscle health, with higher HU values indicating denser and healthier muscle tissue, and lower values suggesting infiltration of fat or other non-muscle tissues [16]. 

We hypothesized that dietary supplementation of MCTs would promote nutritional support of the physical elements of the elderly that regulate balance during walking. Thus, the goal of this study was to investigate the effects of MCTs on the metabolism and quality of gait-related skeletal muscles in healthy older adults.

## 2. Materials and Methods

### 2.1. Study Design and Ethics Approval

This was a randomized, double-blind, placebo-controlled, parallel-group, two-arm (1:1 ratio) trial conducted between October 2018 and December 2019, registered as the MCT SMILE (SuppleMentary for Life in the Elderly) project study (UMIN000033447). The study procedures were performed in accordance with the tenets of the Declaration of Helsinki. The study protocol was approved by the Clinical Research Ethics Committee of the Tohoku University Graduate School of Medicine (2018-2-67, date on 23 July 2018). Written informed consent was obtained from each participant.

### 2.2. Participants

**Figure 1:**
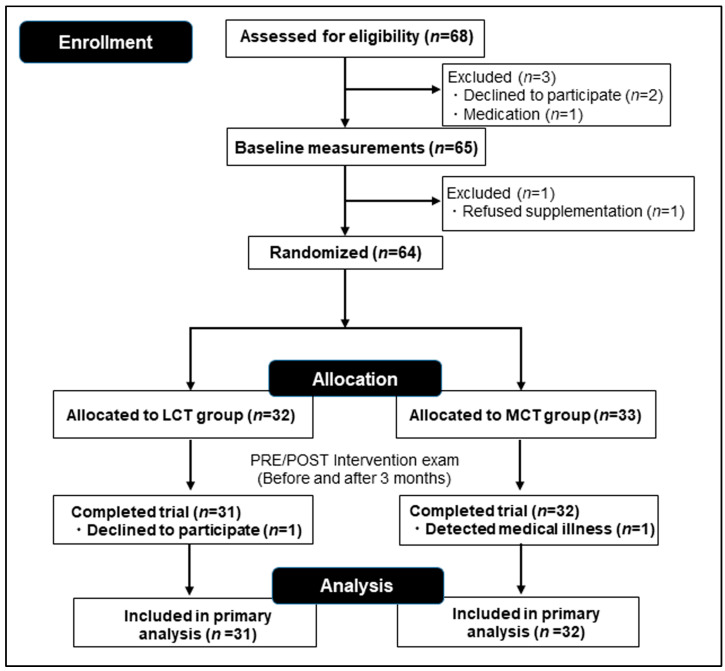
CONSORT diagram describing recruitment and random allocation of the participants.

Details of the original MCT SMILE study have been described previously [10]. Briefly, the inclusion criteria were an age of 65–80 years, right-handed, without dementia or mild cognitive impairment, and with full autonomy in activities of daily living. Participants taking nutritional supplements affecting energy metabolism or body composition were excluded from the study. Eligible participants were assigned randomly to the MCT group or a placebo control group who took a matching formula made with canola oil (LCT) as a caloric equivalent to the MCT-based vegetable oil (Figure 1). Each participant was scheduled for imaging studies, neurocognitive and physical function tests, and blood examinations before and after the 3-month intervention. A yogurt-flavored jelly stick was used (total weight, 15 g) containing 6 g of MCTs (75% C8:0 and 25% C10:0 from total fatty acids) or 6 g of LCT (64% C18:1, 19% C18:2, and 9% C18:3 from total fatty acids) [12]. In this study, participants and researchers could not distinguish between the two supplements from the appearance (created using an unlabeled silver stick), flavor, or texture. Participants were instructed to consume 3 sticks per day, one just prior to every meal, for a total of 3 consecutive months. Lifestyle, eating habits, and medications were not changed throughout the study period. Each participant maintained a daily logbook to ensure their compliance.

### 2.3. Image Acquisition

Whole-body FDG PET/CT images were acquired on a GE Discovery PET/CT scanner (GE Healthcare, Milwaukee, WI, USA). Low-dose CT was performed at 140 kVp, 25 mAs, and a layer thickness of 2 mm. Brain PET images were acquired for 3 min in three-dimensional (3D) mode using a single bed position. The patients were required to fast for a minimum of 12 h before starting the PET scan to ensure the plasma glucose level was ≤6.0 mM. Image acquisition started approximately 60 min after injection of 3.7 MBq/mL/kg ^18^F-FDG. PET images incorporating all image corrections were reconstructed using the manufacturer’s software and recommended parameters (β = 200; field of view, 300 mm; matrix size, 128 × 128; layer thickness, 3.26 mm) to obtain maximum intensity projection and fused images. Imaging data were then imported into LIFEx open-source software (v7.8.0; http://www.lifexsoft.org: accessed on 17 May 2025) in the Digital Imaging and Communications in Medicine (DICOM) format to analyze the PET metabolic and CT density parameters. The SUV_mean_ of each ROI for both sides was calculated using LIFEx software. The mean SUV_mean_ for patient-based assessments was calculated by averaging the values obtained for each unilateral region.

**Figure 2:**
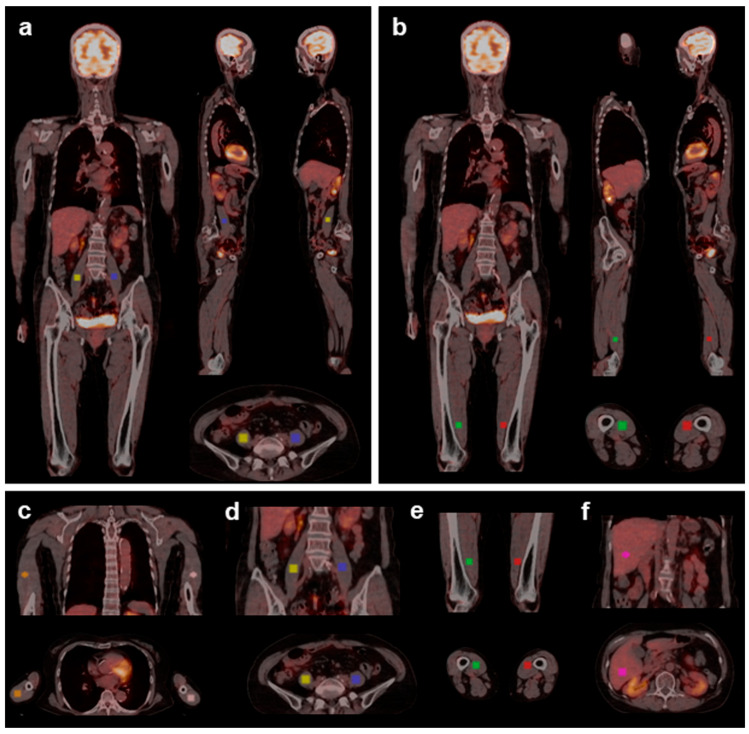
Representative whole-body FDG PET/CT images of ROI placement on the psoas (**a**) and vastus medialis (**b**). Three-dimensional ROIs (5.1 cm^3^) are placed at the widest and thickest part of each muscle belly by confirming the coronal, sagittal, and cross-sectional images. ROI placement on the skeletal muscles and liver: The major gait-related muscles ((**c**), triceps; (**d**), psoas; (**e**), vastus medialis) are identified bilaterally on PET/CT fusion images. The liver ROI is used as the standard reference for glucose uptake (**f**). The upper and lower rows represent coronal and cross-sectional images, respectively. ROI: region of interest.

A 3D ROI of 5.1 cm^3^ was manually placed at the widest and thickest part of each relevant muscle belly identified by the PET/CT fusion image (Figure 2a,b). This study focused on the relationship between muscle density and metabolism of the major large muscles associated with walking balance (bilateral triceps, psoas, and vastus medialis) (Figure 2c–e) rather than changes in muscle quantity (mass) because we aimed to clarify the primary biological effects of MCT on skeletal muscles without muscle atrophy. As a reference and for assessing hepatic FDG phosphorylation, an ROI (3 cm in diameter) was placed in the upper right lobe of the liver (Figure 2f) [17].

### 2.4. Measurements

The PET/CT-derived SUV_mean_ and HU values were measured before and 3 months after starting the intervention. Blood samples were analyzed for metabolic byproducts, including ketone bodies (β-hydroxybutyrate and acetoacetate) and glucose derived from the blood plasma samples, by a commercial clinical laboratory (SRL Inc., Tokyo, Japan) before and 3 months after intervention. The venous blood samples were taken after a 12-h overnight fast through an intravenous catheter secured for the PET study, as described [10].

### 2.5. Statistical Analysis

A minimum sample size of 28 was established from a previous study [18] on the cognitive effects of MCT supplementation in elderly patients, which was calculated to detect a clinically relevant effect with a 2-sided *α* of 0.05 and 90% power. Allowing for a 10% dropout during the intervention, the total required sample size for the present study was estimated as 31 per group. Continuous data are presented as the mean ± standard deviation. Repeated-measures two-way analysis of variance was used to determine differences within each variable. Two time points were included: the within-participant factor (effect per unit of time), and the between-participant factor (the differences between the MCT and control groups). When the group × time interaction was significant, tests of simple effects were performed to determine whether the groups differed significantly during the intervention period, using post hoc analyses adjusted for the Bonferroni correction (α level 0.025), where appropriate. Pearson’s correlation coefficients were calculated to investigate the relationship between ketone body synthesis (β-hydroxybutyrate) and skeletal muscle glucose metabolism (SUV_mean_) before and after intervention. Linear regression analyses were performed to evaluate the SUV_mean_ and muscle density (HU) association within the same ROI for each muscle. Statistical significance was set at *p* < 0.05. All analyses were performed using SPSS version 28 (IBM; Armonk, NY, USA), Bell Curve for Excel (SSRI, Tokyo, Japan), and Prism 9 (GraphPad Software; La Jolla, CA, USA).

## 3. Results

### 3.1. Characteristics of Participants

Among 68 participants, 63 (females/males: 22/10 in the MCT group and 21/10 in the placebo group) completed the study (Figure 1). The baseline demographic data of the 63 participants were not significantly different between the groups (see the demographic data of the participants for the original MCT SMILE study [10]).

### 3.2. Effects of MCT on Blood Ketone Bodies and Glucose Metabolism

MCT supplementation had an effect on maintaining ketone bodies after a 3-month intervention; they decreased in the placebo control group (Table 1). In the MCT group, several weak but significant correlations were found between plasma β-hydroxybutyrate and the SUV_mean_ of gait-related skeletal muscles, represented by the iliopsoas and vastus medialis; no relationships were apparent in the placebo control (Table 2).

**Figure 3:**
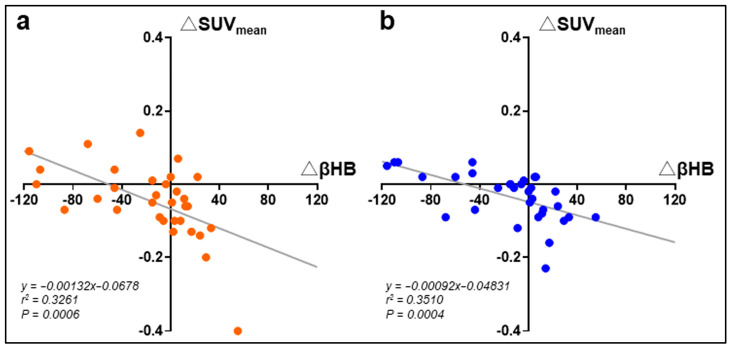
Scatter plots of the changes in plasma β-hydroxybutyrate and mean standardized uptake value (SUV_mean_) of the psoas (**a**) or vastus medialis (**b**) muscles with regression lines in participants after 3-month MCT oil supplementation. βHB: plasma β-hydroxybutyrate.

For the MCT-treated participants, simple linear regression analysis of the longitudinal data measured before and after the intervention suggests that β-hydroxybutyrate was associated negatively with glucose metabolism in these two muscles, which are responsible for balance and posture during walking (Figure 3).

### 3.3. Effects of MCT on Glucose Metabolism and Muscle Density

**Figure 4:**
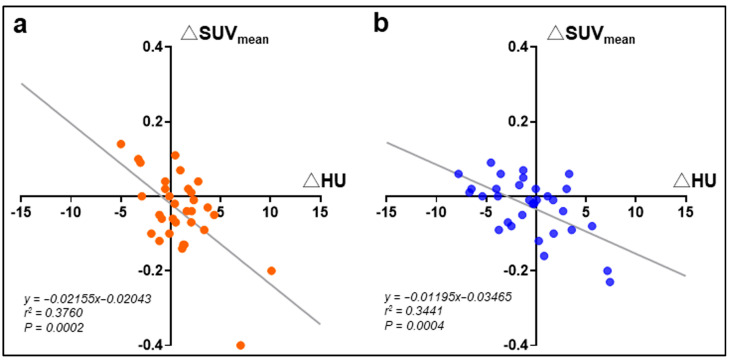
Scatter plots of the changes in CT mean standardized uptake value (SUV_mean_) of the psoas (**a**) or vastus medialis (**b**) muscles with regression lines in participants after a 3-month MCT oil supplementation. βHB: plasma β-hydroxybutyrate.

For post-intervention glucose metabolism, MCT decreased the SUV _mean_ in the lower skeletal muscles (psoas and vastus medialis), with a slight elevation in the liver (Table 3), but no significant changes in blood glucose levels (Table 1). In addition, MCT increased the post-intervention HU of these two muscles, and a significant difference was detected in the psoas muscle compared to the placebo control. For the relationship between glucose metabolism and skeletal muscle density in participants supplemented with MCT, the SUV_mean_ was negatively associated with HU values (*r* = −0.613 [95%CI: −0.793~−0.337], *p* = 0.0002 [Psoas]; *r* = −0.587 [95%CI: −0.777~−0.299, *p* = 0.0004 [Vastus medialis]) (Figure 4). No apparent changes in the metabolic or muscle parameters were detected in the placebo group.

## 4. Discussion

### 4.1. Mechanism by Which MCTs Modulate Energy Metabolism

Increased levels of ketone bodies attenuate glucose utilization in peripheral tissues, have anti-lipolytic effects in adipose tissue, and potentially attenuate proteolysis in skeletal muscles [19]. It is unclear why chronic MCT supplementation did not elevate but rather declined plasma ketone bodies in our participants. However, the sustained circulating β-hydroxybutyrate, the most abundant ketone body [20], observed only in the MCT supplemented participants may reflect certain beneficial metabolic changes due to MCTs. In fact, MCTs are rapidly absorbed and metabolized, converting into ketones with varied estimated plasma half-lives depending on the type (0.8 to 3.1 h for β-hydroxybutyrate and 8 to 14 h for acetoacetate) [21]. Therefore, ketone bodies sampled at least 12 h after the last MCT ingestion could have been reduced compared to their peak plasma levels.

PET image analysis showed glucose hypometabolism in the psoas and vastus medialis after a 3-month MCT intervention, indicating altered energy metabolism and glucose utilization. High-fat ketogenic diets achieved by MCTs have been shown to induce a “metabolic switch” from glycolysis to ketone body utilization in the mitochondrial citric acid cycle, preferentially in the heart, skeletal muscle, and brain tissues in healthy adults [22,23]. These observations can be explained by the inverse relationship between ketolysis and glycolysis in the lower-body skeletal muscles [19] and regional brain areas (e.g., the primary sensorimotor cortex) related to gait balance control through increased cerebellar neural connectivity [10].

### 4.2. Effects of MCTs on Skeletal Muscle Mass and Density

This study acquired whole-body CT fusion images simultaneously with PET scans to evaluate muscle quantity (mass) determined by the skeletal muscle area [24]. However, for healthy older adults without anatomical changes in muscle mass, the quality of the muscle seems more important than the quantity given the relatively short study period (3 months). Normal reference data regarding CT-derived muscle parameters have been reported recently, including skeletal muscle density (HU) [24,25]. As a measure of muscle quality, myosteatosis can be indirectly assessed by muscle radiodensity attenuation in the skeletal muscles of the lower extremities [26]. Notably, MCTs caused slight but significant time-course enhancement of CT densities in the psoas and vastus medialis muscles within the normal range (+30 to +150 HU) [24] and above lower thresholds (psoas, 46.1) [24]. The psoas muscle is important in dynamic function and postural support; the vastus medialis and the quadriceps provide stability during the stance phase in the gait cycle and support normal posture. Therefore, increased HU may affect muscle quality, improving gait function following MCT supplementation.

Unfortunately, segmenting multiple skeletal muscle regions in abdominal or abdominopelvic CT images is difficult for several reasons, including muscle morphology, signal intensity, and image artifacts [27]. Due to this, further image analyses incorporating magnetic resonance imaging data are expected to clarify the MCT-induced differences in regional energy metabolism, especially the reliance on oxidative phosphorylation between slow-twitch (type I) and fast-twitch (type II) fibers and their subtypes, which contribute to long-term endurance and powerful bursts of gait movement, respectively [19].

### 4.3. Practical Application of MCTs

Frailty and sarcopenia are major health issues among elderly people [28]. The potential of MCTs to address these age-related physical conditions is just beginning to be studied since the 2000s. We have recently reported the effect of MCTs on increasing muscle functionality without altering muscle mass in healthy older adults. Supplementation of MCTs with or without aerobic exercise improved muscle functions (e.g., knee extension strength and balance ability during walking) without changing the skeletal muscle mass compared with the placebo control using LCTs [8]. These findings have been confirmed in vivo by detecting altered glucose metabolism both in the brain (e.g., right primary sensorimotor cortex) and in some skeletal muscles responsible for gait motor functions, and increased neural connectivity between brain areas related to balance and memory functions (e.g., contralateral cerebellum to the bilateral amygdala and anterior hippocampus), using FDG-PET/CT and functional magnetic resonance imaging studies [10]. The beneficial aspects of MCTs are not limited to healthy subjects and could also be applicable to frail older adults. It is interesting to note that MCT supplementation at smaller doses (6 g/day for 3 month) has been shown to increase both muscle mass and function and to decrease fat mass with maintained or increased body weight [12], suggesting some positive “add-on” effects of MCTs on anatomical changes associated with frailty/sarcopenia.

### 4.4. Limitations

There are several limitations in our study. First, this study may have been underpowered for detecting the desired group differences because of a small sample size; however, its risk for bias was minimized compared to other RCTs [29]. Second, we could not target ketone body metabolism in skeletal muscles because of limited PET tracer, including ^11^C-β-hydroxybutyrate [30]. Indeed, it is hard to determine the ketogenic shift of each muscle using only the circulating ketone bodies with shorter half-lives. Nevertheless, significant negative relationships between plasma β-hydroxybutyrate levels and imaging outcomes may, at least, support a favorable biological effect of MCTs on selected skeletal muscles responsible for walking in healthy older adults.

## 5. Conclusions

A 3-month MCT supplementation suppressed glucose metabolism in gait-related skeletal muscles and increased their muscle density, consistent with previous findings that it stabilizes balance functions during walking in healthy older adults [10]. Present functional/morphological imaging results extend our knowledge of MCTs’ biological effect against senescent change.

## Figures and Tables

**Table 1:** Changes in plasma ketone bodies before (Pre) and after (Post) intervention.

	Placebo			Medium-Chain Triglycerides			Intergroup *p* Value
	Pre	Post	*p*	Pre	Post	*p*	
**Total ketone bodies (μmol/L)**	155 ± 96	112 ± 78	0.01	110 ± 87	90 ± 62	0.17	0.09
**β-hydroxybutyrate (μmol/L)**	105 ± 65	74 ± 53	0.03	74 ± 61	58 ± 46	0.46	0.05
**Acetoacetate (μmol/L)**	50 ± 33	38 ± 26	0.006	36 ± 28	32 ± 17	0.10	0.08
**Glucose (mmol/L)**	5.3 ± 0.3	5.3 ± 0.3	0.57	5.4 ± 0.5	5.5 ± 0.4	0.06	0.06

Data are expressed as the mean ± standard deviation. Statistically significant results with *p* < 0.05 are shown in bold.

**Table 2:** Pearson correlation coefficient between plasma β-hydroxybutyrate and gait-related skeletal muscle SUV_mean_ before and after intervention.

	Placebo			Medium-Chain Triglycerides		
	*r*	*95%CI*	*p*	*r*	*95%CI*	*p*
**Triceps**	−0.102	−0.446 0.268	0.59	−0.341	−0.617 0.008	0.056
**Psoas**	−0.029	−0.385 0.335	0.88	−0.571	−0.767 −0.278	0.0006
**Vastus medialis**	0.026	−0.337 0.383	0.89	−0.593	−0.780 −0.307	0.0004

Statistically significant results with *p* < 0.05 are shown in bold. CI: confidence interval.

**Table 3:** PET metabolic and CT density parameters before (Pre) and after (Post) intervention.

	Mean Standardized Uptake Value			Intergroup *p* Value	Hounsfield Units			Intergroup *p* Value
	Pre	Post	*p*		Pre	Post	*p*	
**Triceps**								
Placebo	0.64 ± 0.11	0.62 ± 0.08	0.07	0.98	60.5 ± 7.3	59.6 ± 7.9	0.36	0.06
MCT	0.63 ± 0.10	0.63 ± 0.09	0.80		63.4 ± 6.4	63.1 ± 7.8	0.73	
**Psoas**								
Placebo	0.60 ± 0.12	0.59 ± 0.10	0.51	0.43	54.2 ± 4.1	53.1 ± 2.9	0.30	<0.001
MCT	0.63 ± 0.08	0.59 ± 0.10	0.002		49.8 ± 3.8	53.0 ± 3.9	0.002	
**Vastus medialis**								
Placebo	0.60 ± 0.10	0.61 ± 0.10	0.67	0.69	46.5 ± 6.0	44.3 ± 5.1	0.15	0.11
MCT	0.63 ± 0.09	0.60 ± 0.08	0.03		42.4 ± 6.5	45.4 ± 4.8	0.04	
**Liver**								
Placebo	2.28 ± 0.31	2.29 ± 0.37	0.64	0.97	62.6 ± 6.4	64.3 ± 6.8	0.05	0.95
MCT	2.23 ± 0.39	2.35 ± 0.34	0.003		63.3 ± 5.9	63.4 ± 7.0	0.89	

Data are expressed as the mean ± standard deviation. Statistically significant results with *p* < 0.05 are shown in bold. PET: positron emission tomography, CT: computed tomography, MCT: medium-chain triglycerides.

## Data Availability

As this study contains only a small number of participants, some of the personal information that may assist in the deduction or reveal the identity of participants has been deleted in order to protect the privacy of these individuals.

## Abbreviations

The following abbreviations are used in this manuscript:

CT	Computed Tomography
FDG	^18^F-2-fluoro-2-deoxy-D-glucose
HU	Hounsfield units
LCT	Long-chain triglyceride
MCT	Medium-chain triglyceride
PET	Positron emission tomography
RCT	Randomized clinical trial
ROI	Region of interest
SUV	Standardized uptake value
3D	Three-dimensional.
